# Improvements in the Quantitative Assessment of Cerebral Blood Volume and Flow with the Removal of Vessel Voxels from MR Perfusion Images

**DOI:** 10.1155/2013/382027

**Published:** 2013-03-18

**Authors:** Michael Mu Huo Teng, I-Chieh Cho, Yi-Hsuan Kao, Chi-Shuo Chuang, Fang-Ying Chiu, Feng-Chi Chang

**Affiliations:** ^1^School of Medicine, National Yang Ming University, Taipei 112, Taiwan; ^2^Department of Radiology, Taipei Veterans General Hospital, Taipei 112, Taiwan; ^3^Department of Biomedical Imaging and Radiological Sciences, National Yang-Ming University, No. 155, Section 2, Li-Nong Street, Bei-Tou, Taipei 112, Taiwan

## Abstract

*Objective*. To improve the quantitative assessment of cerebral blood volume (CBV) and flow (CBF) in the brain voxels from MR perfusion images. *Materials and Methods*. Normal brain parenchyma was automatically segmented with the time-to-peak criteria after cerebrospinal fluid removal and preliminary vessel voxel removal. Two scaling factors were calculated by comparing the relative CBV and CBF of the segmented normal brain parenchyma with the absolute values in the literature. Using the scaling factors, the relative values were converted to the absolute CBV and CBF. Voxels with either CBV > 8 mL/100 g or CBF > 100 mL/100 g/min were characterized as vessel voxels and were excluded from the quantitative measurements. *Results*. The segmented brain parenchyma with normal perfusion was consistent with the angiographic findings for each patient. We confirmed the necessity of dual thresholds including CBF and CBV for proper removal of vessel voxels. The scaling factors were 0.208 ± 0.041 for CBV, and 0.168 ± 0.037, 0.172 ± 0.037 for CBF calculated using standard and circulant singular value decomposition techniques, respectively. *Conclusion*. The automatic scaling and vessel removal techniques provide an alternative method for obtaining improved quantitative assessment of CBV and CBF in patients with thromboembolic cerebral arterial disease.

## 1. Introduction

Dynamic-susceptibility-contrast MR imaging is used to diagnose cerebral blood perfusion. Parametric measurements, such as cerebral blood volume (CBV), cerebral blood flow (CBF), mean transit time (MTT), and time-to-peak (TTP), are used to evaluate local perfusions [1–5]. Quantitative evaluation of these parametric images provides valuable information on the deficiencies in local brain perfusions [6–10]. An accurate diagnosis of the location and severity of decreased brain perfusion is crucial for proper treatment, as brain tissues with delayed perfusion can indicate a deteriorating condition [11–13].

The CBV and CBF of the brain parenchyma are usually overestimated in MR perfusion compared to computed tomography (CT) and positron emission tomography (PET) (Table 1). There are several factors that may cause overestimation of CBV and CBF in MR brain perfusions. First, it is difficult to find an arterial voxel consisting of 100% blood for the CBV and CBF calculations because the diameter of an artery is smaller than the voxel size of 1.6 × 1.6 × 7 mm^3^ used in MR perfusion imaging. Second, although the concentration-time curve measured in a venous voxel has been proposed to correct for the partial volume of blood in arterial voxels [14, 15], the assumption that at least one venous voxel consists of 100% blood is not guaranteed for all MR brain perfusion studies [14, 16]. Third, the phase between blood and tissues can cause signal cancellation in the voxels that consist of both blood and tissues [17]. This phase effect is TE dependent and can cause unexpected signal changes in arterial voxels that do not consist of 100% blood. Fourth, the changes in MR relativity are different in tissues and blood [18, 19]. Fifth, The CBV and CBF in blood vessels are much higher than those in the brain parenchyma. The measured CBV and CBF are variable with inclusion of different amount of vessel voxel in the manually drawn regions of interest (ROIs). 

To achieve an absolute calculation of the CBV and CBF, Østergaard et al. suggest the use of a scaling factor [5] in which the relative CBV (rCBV) of normal white matter is compared with a standard value of 2.1 mL/100 g from a PET study [23]. To identify the normal white matter for calculation of the scaling factor, Mukherjee et al. propose a manual drawing technique [24]. Sakaie et al. also develop a manual drawing technique for comparing the rCBV of white matter with absolute values measured on steady-state CBV images, on a subject-by-subject basis [22]. However, the manual drawing technique is user dependent, and it is difficult to identify white matter with normal perfusion in some patients. 

In this study, we established an automatic segmentation technique for identifying normal brain parenchyma. Two scaling factors were calculated by comparing the rCBV and relative CBF (rCBF) of the segmented normal brain parenchyma with reference values. The absolute CBV and CBF of the images were calculated with the two scaling factors. The vessel voxels with high CBV and CBF values were removed to improve the quantitative measurements of the brain parenchyma. 

## 2. Materials and Methods

### 2.1. Image Acquisition

Clinical MR perfusion images were routinely acquired from patients with cerebral vascular diseases using a 1.5-Tesla MR imager in our hospital. We retrospectively analyzed perfusion images acquired from 30 subjects who received either a half or full dose of the contrast agents (Table 2). For the half-dose group, Gd-DOPTA (MultiHancet, Bracco Imaging SpA, Milan, Italy) was used at a dose of 0.05 mM/kg body weight. For the full-dose group, Gd-DTPA (Magnevist, Schering, Germany) was used at a dose of 0.1 mM/kg body weight. Each group included 6 subjects with good collateral blood flow on the side of carotid stenosis and 6 subjects with poor brain perfusion that was due to severe carotid stenosis or occlusion with poor collateral circulation from the circle of Willis or M1 occlusion. In addition, 6 subjects with normal internal carotid and middle cerebral arteries were included in the half-dose group.

A single-shot, gradient-echo echo-planar imaging technique was used to obtain the perfusion images. The scan parameters were as follows: TR = 1000 ms, TE = 40 ms, flip angle = 60 degrees (half-dose group) or 90 degrees (full-dose group), field of view = 24 × 24 cm^2^, k-space matrix size = 128 × 80 (EPI factor = 80), and image matrix size = 128 × 128. The slice thickness was 7 mm with a gap of 7 mm. Seven slices were acquired with 70 dynamic images, and the temporal resolution was 1s. For both groups, the injection rate of 3 mL/s was achieved with a power injector (Spectris, Medrad, Indianola, PA). The local Institutional Review Board approved this study. Written informed consent was obtained from each patient in accordance with our routine clinical protocols. Image postprocessing was performed using software written in MatLab (Mathworks, Natick, MA).

### 2.2. Parametric Image Calculations

A brain mask was produced by applying Otsu's thresholding technique [25] to the first perfusion image to remove signals outside the brain surface. The concentration-time curve, *C*(*t*), for a voxel included in the brain mask was calculated as follows:

(1) $$\begin{array}{c} C\left(t\right)=\frac{k_{1}}{\text{TE}}\ln\left(\frac{S\left(t\right)}{S_{0}}\right), \end{array}$$

where *k*_1_ is a proportionality factor, TE is the echo time, *S*(*t*) is the signal at time *t*, and *S*_0_ is the baseline signal calculated as the average of the 6th through the 10th perfusion images [1–5]. The rCBV was calculated as the summation of the concentration-time curve from the 11th to the 70th temporal points, described as

(2) $$\begin{array}{c} \text{rCBV}=\frac{k_{H}}{\rho }\cdot \frac{\sum_{t=11}^{70}C\left(t\right)}{\sum_{t=11}^{70}\text{AIF}\left(t\right)}, \end{array}$$

where AIF(*t*) is an arterial input function calculated as the average concentration-time curve of the arterial voxels. The independent component analysis technique was used to select arterial voxels on the normal side of the brain to measure AIF(*t*) [26]. Values of *ρ* = 1.04 g/cc, *k*_*H*_ = 0.73, and *k*_*H*_/*ρ* = 0.705 cc/g were used in this study [3]. To calculate the rCBF, the concentration-time curve at a tissue voxel was expressed as a convolution process, described as

(3) $$\begin{array}{c} C_{\text{tissue}}\left(t\right)=\frac{\rho }{k_{H}}\cdot \text{AIF}\left(t\right)⊗\left[\text{rCBF}\cdot R\left(t\right)\right], \end{array}$$

where *R*(*t*) is a residue function describing a local perfusion pattern. The standard and circulant singular value decomposition (sSVD) (cSVD) techniques [4, 5, 27, 28] were used to perform the deconvolution calculation to obtain the [rCBF · *R*(*t*)] curve [4, 5]. The maximum value of the [rCBF · *R*(*t*)] curve was used as the rCBF.

### 2.3. Cerebrospinal Fluid Voxel Removal

**Figure 1:**
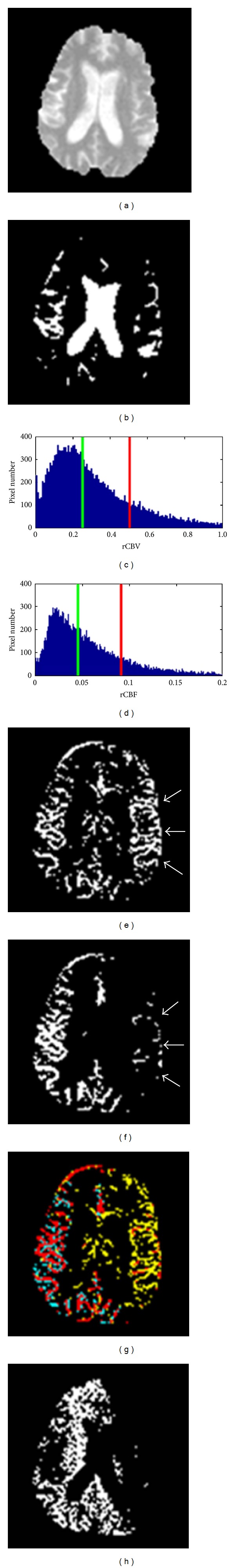
The postprocessing procedure from a patient with 90% left internal carotid stenosis (patient #1 in Table 2). A ratio image (a) and CSF mask (b) were obtained for CSF removal. Histograms of the rCBV (c) and the rCBF (d) after CSF removal were plotted. The median values of the rCBV and rCBF are marked with green lines. The thresholds for the preliminary vessel voxel removal were set at twice the median (red lines). Preliminary vessel masks were calculated from the rCBV image (e), rCBF image (f), and both rCBV and rCBF images (g). In (g), yellow voxels are from the rCBV mask, cyan voxels are from the rCBF mask, and red voxels are from both. More vessel voxels are identified on the stenotic side (arrows) than the normal side on rCBV mask (e). Vessel voxels on the lesion side (arrows) are not properly demonstrated on rCBF mask (f). The segmented normal perfused brain parenchyma is shown in (h).

To improve the outline of the brain parenchyma and to reduce the interference of the cerebrospinal fluid (CSF) spaces and their contents, CSF voxels were removed using a previously reported technique [29]. An example (case #1) is shown in Figures 1(a) and 1(b). A ratio image was calculated by dividing the first image by the baseline image (the average of the 6th through the 10th perfusion images) (Figure 1(a)). By applying a threshold to the ratio image, the CSF voxels (Figure 1(b)) were identified and removed on the parametric images. There was some overlapping of signal intensity between CSF and the brain parenchyma in the first ratio image because of partial volume effect. To avoid individual variability in determining the threshold for CSF removal, we adopt the results obtained by Otsu's thresholding technique [25]. 

### 2.4. Preliminary Vessel Voxel Removal and Normal Brain Segmentation

After removing the CSF voxels, the median values of the rCBV and rCBF (green lines in Figures 1(c) and 1(d)) were calculated from the remaining brain voxels. Twice of the median values were used as the thresholds for segmenting vessel voxels (Figures 1(e) and 1(f)). Voxels with an rCBV or rCBF higher than the established threshold were preliminarily classified as vessel voxels and were removed from the brain mask (Figure 1(g)). The purpose of this preliminary vessel removal procedure was to help with identifying normal brain parenchyma. Brain parenchyma with normal perfusion (Figure 1(h)) was segmented by selecting voxels with TTP values between the (TTP_median_ − 3 sec) and TTP_median_, where the TTP_median_ is the median of the TTP. 

### 2.5. CBV and CBF Scaling

We assumed that (1) normal brain parenchyma consists of 60% gray matter and 40% white matter, and (2) the average rCBV and rCBF in the normal brain parenchyma were linearly correlated with the absolute CBV and CBF values reported in the literature. Two scaling factors were calculated to convert the measured rCBV and rCBF to absolute values of CBV and CBF. According to studies using PET and CT (Table 1), the0 CBV for normal white matter and normal gray matter are approximately 2 and 4 mL/100 g, respectively. We used the average rCBV in the segmented normal brain and a standard CBV value of 3.2 mL/100 g to calculate a scaling factor (SF_CBV_) to convert the rCBV image to the absolute CBV ( = SF_CBV_ × rCBV) image. Similarly, from previous PET and CT studies, the CBF values for normal white matter and normal gray matter are approximately 25 and 50 mL/min/100 g, respectively (Table 1). We used the average rCBF in the segmented normal brain and the standard CBF of 40 mL/min/100 g to calculate a scaling factor (SF_CBF_) to convert the rCBF image to the absolute CBF ( = SF_CBF_ × rCBF) image. After calculating these two scaling factors, the CBV and CBF values were calculated from the rCBV and rCBF values for all voxels inside the brain mask determined by using the Otsu's technique. The MTT value was calculated MTT = CBV/CBF.

### 2.6. Final Vessel Voxel Removal

For the final CBV, CBF, and MTT images, we adopted the vessel removal threshold of CBV > 8 mL/100 g or CBF > 100 mL/100 g/min that was reported by Murphy et al. [30]. The vessel pixels obtained from CBV and CBF thresholds were compared. The rCBV and rCBF thresholds used in the preliminary vessel removal procedure were converted to the absolute CBV and CBF values for comparison.

### 2.7. Measurements Using ROIs

To measure the rescaled CBV, CBF, and MTT of brain parenchyma, we placed a hemisphere-sized ROI over each hemisphere at the plane containing the bodies of the lateral ventricles. The mean and standard deviation was measured four times, as follows: (1) all voxels inside the brain mask determined by using the Otsu's thresholding technique, without removal of the CSF or vessels; (2) the brain mask after CSF voxel removal; (3) the brain mask after vessel voxel removal; and (4) the brain mask after removal of both the CSF and vessel voxels.

## 3. Results

**Figure 2:**
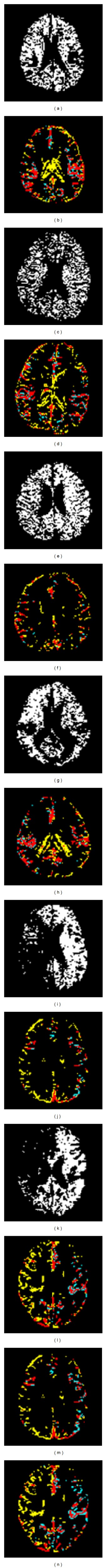
Representative images of the segmented normal brain parenchyma ((a), (c), (e), (g), (i), (k)) and vessel masks generated from CBV images and CBF images calculated using the cSVD ((b), (d), (f), (h), (j), (l)) and sSVD ((m), (n)) techniques in the final vessel removal step. In the color-coded vessel masks, yellow voxels are detected with a CBV threshold (CBV > 8 mL/100 g) only, cyan voxels are found from the CBF threshold (CBF > 100 mL/100 g/min) only, and red voxels are found from both. Different degrees of stenosis are shown in this figure: bilateral normal arteries (0% stenosis): subject #14 ((a) and (b)); 40% stenosis (right side): subject #11 ((c) and (d)); 75% stenosis (right side): subject #26 ((e) and (f)); 90% stenosis (left side): subject #29 ((g) and (h)); 90% stenosis (right side): subject #23 ((i), (j), and (m)); 95% stenosis (right side): subject #20 ((k), (l), and (n)). In patients with unilateral high-grade stenosis ((j), (l), (m), (n)), the CBV threshold (yellow + red) detected insufficient vessels on the normal side, and the cSVD CBF masks ((j), (l)) and sSVD CBF masks ((m), (n)) (cyan + red) detected insufficient vessels on the lesion side.

The segmented brain masks with normal perfusion were consistent with the patients' arterial and clinical conditions in all patients in this study (Figure 2). The bilateral hemispheres in the normal brain mask are grossly symmetrical in patients with bilateral normal arteries (Figure 2(a)) or unilateral slight stenosis (Figure 2(c)—right side, 40%). The normal brain mask is slightly asymmetrical in patients with unilateral moderate stenosis (Figure 2(e)—right side, 75%) or unilateral severe carotid stenosis with good collateral circulation (Figure 2(g)—left side, 90%). The normal brain masks are obviously asymmetrical, with no or few voxels on the side with severe unilateral carotid stenosis and poor collateral circulation at the Willis' circle or severe unilateral stenosis at M1 (Figure 2(i)—right side, 90%; Figure 2(k)—right side, 95%). 

Representative images of the final vessel mask are shown in Figure 2. The choroid plexus in the lateral ventricle were identified mainly in CBV-based vessel mask (in yellow) and not in the CBF-based mask (Figures 2(b), 2(d), and 2(h)) because of high CBV and low CBF in the choroid plexus. In patients with high-grade stenosis and poor collateral circulation, vessel voxels on the lesion side were mainly detected by the CBV threshold (yellow + red) and insufficiently detected by the CBF threshold (cyan + red). On the contrary, vessel voxels on the normal side were mainly detected by the CBF threshold (cyan + red) and insufficiently detected by the CBV threshold (yellow + red). The vessel masks calculated by sSVD are similar to cSVD vessel masks (Figures 2(b), 2(d), 2(f), 2(h), 2(j), and 2(l)). Only in patients with high grade stenosis and poor collateral circulation, the insufficient detection of vessel voxels on the normal side of the brain on CBV-based mask (yellow + red) was more obvious. The sSVD CBF-based mask (cyan + red) detected more vessel voxels on the normal side of the brain (Figures 2(m) and 2(n)) as compared to that detected on cSVD CBF-based mask (cyan + red) (Figures 2(j) and 2(l)). 

The mean and standard deviation of the SF_CBV_ for 30 subjects were 0.208 ± 0.041. The SF_CBF_ values were 0.168 ± 0.037  and 0.172 ± 0.037  for the sSVD and cSVD techniques, respectively. The mean and standard deviation of the absolute values of the preliminary vessel threshold for rCBV (twice the median of the rCBV) was 7.24 ± 0.39 mL/100 g. The absolute values for the rCBF threshold were 83.2 ± 5.0 mL/100 g/min and 85.0 ± 4.7 mL/100 g/min for the sSVD and cSVD techniques, respectively. 

**Figure 3:**
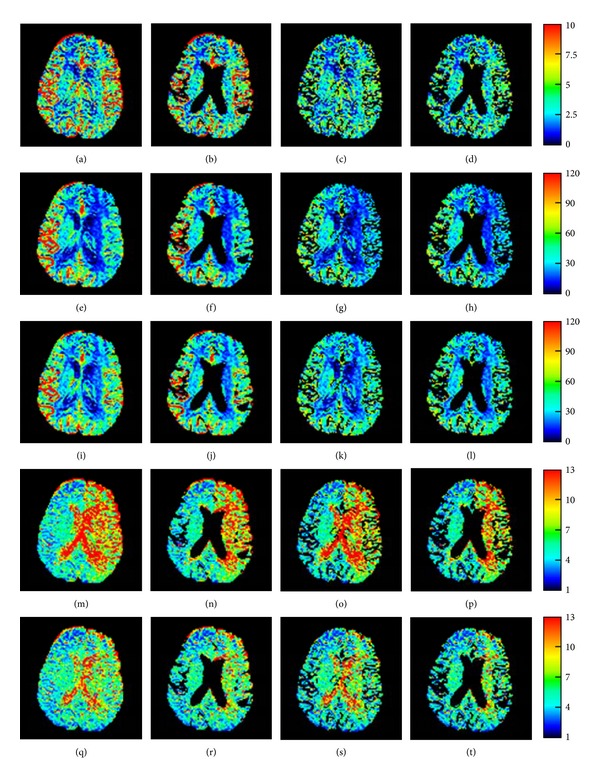
Case 1. Rescaled CBV images (first row), rescaled CBF images calculated using the sSVD (second row) and cSVD (third row) techniques, and rescaled MTT images calculated using the sSVD (fourth row) and cSVD (fifth row) techniques. The images are displayed using the whole brain mask before the removal of the CSF and vessel voxels (first column), after the removal of the CSF voxels (second column), after the removal of the vessel voxels (third column), and after the removal of both the CSF and vessel voxels (fourth column).

**Figure 4:**
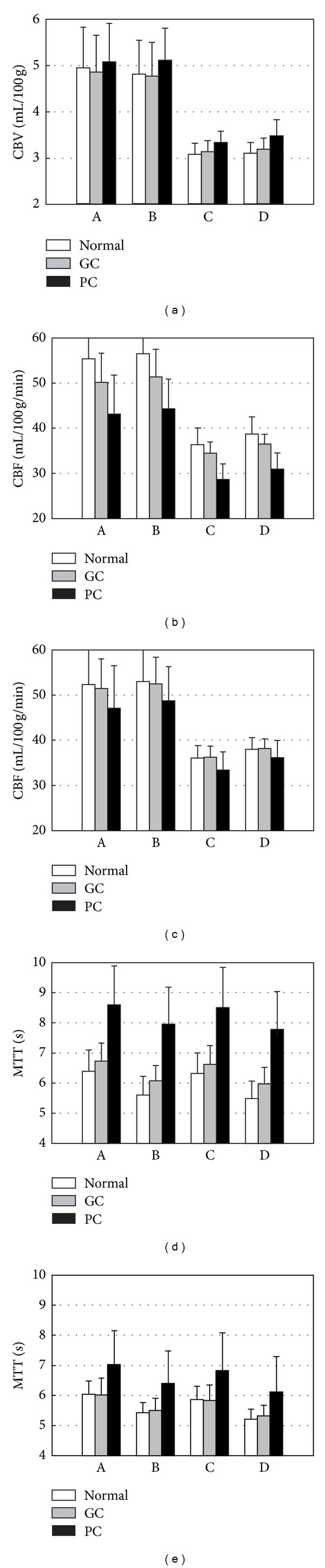
Bar charts are used to present measurements of the CBV (a), CBF ((b), (c)), and MTT ((d), (e)) of the brain parenchyma. The bar charts in (b) and (d) are calculated using the sSVD technique, and the bar charts in (c) and (e) are calculated using the cSVD technique. A total of 60 ROIs were used in the measurements. The results are separated into three groups. The normal group consists of 36 ROIs measured from the normal side of the 30 patients. The good collateral (GC) circulation group consists of 12 ROIs measured from the stenotic side of patients with good collateral blood flow (patients #7~12 and #25~30). In the poor collateral (PC) circulation group, the 12 ROIs were placed on the stenotic side for patients with poor collateral blood flow (patients #1~6 and #19~24). In the measurements, (A) all voxels are included, (B) the CSF voxels are removed, (C) the vessel voxels are removed, and (D) both the CSF and vessel voxels are removed.

An example of the rescaled CBV, CBF, and MTT images (case #1) is shown in Figure 3. Quantitative CBV, CBF, and MTT measurements in the hemispheric ROIs at the level of the body of the lateral ventricles on rescaled images in the 30 patients are presented in Figure 4. The mean and standard deviation of the measured data of the normal brain parenchyma in 30 patients are listed in Table 3. 

The following observations are found in the normal brain parenchyma (Table 3): (1) the CBV and CBF showed no significant change after CSF removal; (2) the MTT was reduced significantly after CSF removal; (3) the CBV and CBF were greatly reduced after the removal of vessel voxels; (4) there was a significant difference in CBV, CBF, and MTT before and after removal of both CSF and vessel voxels; and (5) the absolute values of brain perfusion in the normal brain hemisphere after these post-processing techniques were relatively constant with small variation. The above first four findings were also noted in Figure 4. Comparing the lesion to normal difference calculated from sSVD (Figures 4(b) and 4(d)) and cSVD (Figures 4(c) and 4(e)), there was more obvious difference in CBF and MTT calculated from sSVD in both (1) good collateral group and (2) severe carotid stenosis or occlusion with poor collateral circulation from the circle of Willis or M1 occlusion, as compared to the normal sides. 

## 4. Discussion

In this study, we proposed the use of several techniques to improve the quantification of MR perfusion, including CSF removal, preliminary vessel voxel removal using both the rCBF and rCBV thresholds, automatic segmentation of the brain with normal perfusion, the use of two separate scaling factors to convert the rCBF and rCBV to absolute CBF and CBV data, and final vessel voxel removal using the absolute data of both the CBF and CBV thresholds.

Theoretically, the CBV and CBF should be zero in CSF. After the removal of CSF spaces in the ROI, the CBV and CBF should be increased. However, we found no significant change of CBV and CBF before and after CSF removal (Table 3 and Figure 4). The reason is that the CSF space has overwhelming high signal in the first source image of MR perfusion used for calculating the CSF mask. The small vessels inside the cortical CSF spaces [29] and choroid plexus inside the ventricle (Figures 2(b), 2(d), and 2(h)) were included in the CSF mask. As a result, the CBV and CBF in the CSF mask were not zero and there were no significant changes in CBV and CBF with the CSF removal. Although there was no significant change in the CBV and CBF values, CSF removal improved the outline of the brain parenchyma (Figure 3). With a lack of interference of the CSF space and its contents, we were better able to understand the perfusion changes in the brain parenchyma. 

The quantitative measurements of the CBV and CBF in the brain parenchyma improved with the removal of the vessel voxels on CT perfusion images [20, 21, 30]. The magnetic susceptibility effects of the vessels exaggerate that and extend outside of the vessel voxels may affect the measurement of perfusion of the brain parenchyma [17, 31]. The extravascular high CBV and CBF because of the susceptibility effect will increase the measured values of CBF and CBV of the brain parenchyma if vessel voxels were not removed. Vessel voxel removal resulted in an increased correlation between the MR rCBF and SPECT CBF [32], an increased reproducibility of the MR rCBF measurements [33], and an improved correlation between the MR and PET measurements of the CBF and CBV [9]. Therefore, vessel voxel removal is an important step in the absolute quantification of MR perfusion. 

Only one threshold was used for vessel removal in previous MR perfusion studies. Carroll et al. [33] used the minimum rCBV in a representative vessel area, Grandin et al. used the mean rCBV plus 1 standard deviation [9], and Ernst et al. applied a threshold of 2.5 times the median of rCBF values for all voxels [32]. Only Murphy et al. [30] used both the CBV and CBF thresholds for vessel voxel removal in CT perfusion studies. 

We found that the vessel voxels identified in the rCBV (or CBV) and rCBF (or CBF) from MR perfusion were not the same in some patients with unilateral carotid stenosis (Figures 1(g) and 2). In patients with unilateral severe stenosis and poor collateral circulation, the CBV threshold detected insufficient vessels on the normal side, and the sSVD CBF masks and cSVD CBF masks detected insufficient vessels on the lesion side (Figures 1(g), 2(j), 2(l), 2(m), and 2(n)). This could be explained by the fact that vessels and brain parenchyma on the lesion side in significant impaired perfusion had increased CBV and decreased CBF. Therefore, this study confirmed the necessity of using dual thresholds, one CBF threshold and one CBV threshold, for the proper removal of vessel voxels. 

In the preliminary vessel voxel removal, we used twice the median of the rCBV and twice the median of the rCBF, calculated from the remaining brain voxels after CSF removal, as the thresholds. The mean and standard deviation of the absolute values of the preliminary vessel threshold for rCBV was 7.24 ± 0.39 mL/100 g, which was close to the final vessel voxel removal threshold (8 mL/100 g), and was only 9.5% less. The absolute values for the rCBF threshold were close to each other using sSVD (83.2 ± 5.0 mL/100 g/min) and cSVD (85.0 ± 4.7 mL/100 g/min) techniques and were about 17% to 15% less than the final vessel voxel removal threshold of 100 mL/100 g/min. This means that at the initial step of vessel voxel removal threshold using the criteria (twice the median of the rCBV and twice the median of the rCBF) was smaller, and more vessel voxels were detected and removed as blood vessels. Because the preliminary vessel voxel removal is only a preparation step for segmentation of brain tissue with normal perfusion, it is acceptable that the absolute values of the thresholds were different from the absolute vessel voxel removal thresholds at the final CBV and CBF image demonstration. 

Two scaling factors were used to convert the rCBV and rCBF to absolute values. Only one scaling factor calculated from the rCBV of white matter was used to convert both the rCBV and rCBF to absolute values in previous studies [22, 24]. The use of one scaling factor may not be appropriate, for the following reasons: (1) the rCBV can be overestimated or underestimated, depending on the recirculation patterns in the arterial and tissue voxels, and (2) the rCBF is generally underestimated using the singular value decomposition calculation because of delays in the influx of contrast agents between the arterial and tissue voxels.

Currently the vast majority of application of perfusion study is for cerebral thromboembolic disease. TTP usually is prolonged when there is deficient cerebral arterial blood supply from arterial thromboembolic disease. The described method mainly provides the application of MR perfusion in cerebral thromboembolic disease. Not all brain voxels with TTPs longer than TTP_median_ are abnormal. In this study we arbitrarily used TTP_median_ as the cut-point of normal perfused brain parenchyma. Using TTP value of voxels between (TTP_median_ − 3 sec) and TTP_median_ as the criteria for the segmentation of brain tissue with normal perfusion after CSF removal and preliminary vessel removal, the segmented normal brain parenchyma was compatible with the clinical and angiographic findings in all 30 cases. The described normal perfused brain parenchyma detection technique can also be performed to serve as an instruction for manual ROI placement in the normal white matter for calculation of scaling factor in Mukherjee's technique [24]. 

The benefits of our proposed technique are as follows: (1) the interobserver and intraobserver variability associated with identifying brain tissue with normal perfusion can be eliminated by using the automatic segmentation technique; (2) the individual differences in the CBV, CBF, and MTT of the normal brain parenchyma can be normalized, and a quantitative comparison can be achieved; (3) there is no need to use venous output function to correct for the partial volume effect.

The proposed scaling technique relies on the assumption that the average rCBV and rCBF values of the normal brain parenchyma in patients correspond to the predetermined values. The possible drawbacks of this assumption are as follows: (1) the ratio of voxels containing gray and white matter in the segmented normal brain may vary; (2) the CBV and CBF in the normal brain parenchyma are dependent on age and gender, and different standard values may be used in the future if normal CBV and CBF data for different age groups and their respective genders are reported [23, 34]; and (3) this method may not be applicable to global or diffuse alterations in CBV and/or CBF from aging or neurodegenerative diseases, that lead to changed mean CBF and/or CBF values compared with normal controls. 

Venous output function rescaling [14–16] and the Bookend technique [22] rely on individual correction factors and thus are applicable to individuals with global or diffuse alterations in CBV and/or CBF. However, venous output function rescaling often is not reliable for MR perfusion [14–16]. The Bookend technique provides an individual correction of MR CBV, but CBF correction factor is not separately calculated [22]. Currently the vast majority of application of perfusion study is for cerebral thromboembolic disease. The described method is still valuable and provides the application of MR perfusion in cerebral thromboembolic disease. A recent study using a similar scaling technique showed acceptable linear correlation and quantitative agreement comparing the measured data from MR perfusion and CT perfusion in patients with unilateral occlusion or stenosis of >79% at the internal carotid artery or the middle cerebral artery [35]. 

## 5. Conclusion

We developed an automatic postprocessing technique to improve the quantitative assessment of the cerebral volume and flow in MR perfusion. According to this study, dual thresholds, from both CBV and CBF, are necessary for proper vessel voxel removal. The automatic segmentation of the brain with normal perfusion avoids the potential user variability and difficulty caused by manual selection of normal brain parenchyma. The segmented brain parenchyma with normal perfusion was compatible with the clinical and angiographic condition, in these patients. Using the proposed technique, the standard deviations of the measurements for the CBV, CBF, and MTT were reduced. This technique provides an alternative method for obtaining improved quantitative measurements of the CBV and CBF in the brain parenchyma in patients with thromboembolic cerebral arterial disease.

## Figures and Tables

**Table 1:** Previously reported literature values of the CBV and CBF of normal gray and white matter measured using various imaging modalities.

Modality	CBV (mL/100 g)		CBF (mL/100 g/min)		
	GM	WM	GM	WM	Reference
PET	4.5	2.7	47	20	[8]
PET	3.7	1.3	45	17	[9]
PET			51	38	[20]
CT, VPE	3.9–5.3		51–71		[21]
CT, VPE			53	30	[20]
CT, VPE			42	13	[15]
MR	8.0	4.2	70	34	[3]
MR	6.8	3.8	69	36	[7]
MR	6.0	2.4	59	18	[9]
MR, VOF	9.1	4.6	64	21	[8]
MR, VOF			68	27	[14]
MR, VOF			57	25	[15]
MR, SS	3.7	1.8	57	18	[22]

CT: perfusion CT, MR: dynamic-susceptibility-contrast perfusion MRI, PET: H_2_^15^O positron emission tomography, GM: normal gray matter, WM: normal white matter, VPE: vascular pixel elimination, VOF: partial volume correction using a venous output function, and SS: CBV and CBF scaling using steady-state CBV images.

**Table 2:** Patients' demographic information.

No.	Dose	Collateral circulation	Sex	Age	Stenosis
1	H	P	M	80	LIC (90%)
2	H	P	M	41	LIC (90%)
3	H	P	F	70	RIC occlusion
4	H	P	M	86	LIC (80%)
5	H	P	M	76	RIC (80%)
6	H	P	M	71	LIC stenosis
7	H	G	M	79	RIC (60%)
8	H	G	M	67	RIC (65%)
9	H	G	M	52	RIC occlusion
10	H	G	M	85	LIC (75%)
11	H	G	M	59	RIC (40%)
12	H	G	M	78	LIC (60%)
13	H	N	M	81	Normal
14	H	N	F	81	Normal
15	H	N	M	79	Normal
16	H	N	M	79	Normal
17	H	N	F	83	Normal
18	H	N	M	61	Normal
19	F	P	M	58	LIC (95%)
20	F	P	M	75	RIC (95%)
21	F	P	M	75	LIC (90%)
22	F	P	M	82	RIC (90%), M1 (95%)
23	F	P	M	69	RIC (90%), M1 (70%)
24	F	P	F	66	LIC (90%), M1 (95%)
25	F	G	M	78	RCC (70%)
26	F	G	M	76	RIC (75%)
27	F	G	M	78	RIC (75%)
28	F	G	M	79	RIC (70%)
29	F	G	M	70	LIC (90%)
30	F	G	M	75	RIC (75%)

The abbreviations used are H: half dose, F: full dose, P: poor collateral circulation, G: good collateral circulation, N: no carotid or M1 stenosis, M: male, F: female, RIC: right internal carotid, RCC: right common carotid artery, LIC: left internal carotid, and M1: horizontal segment of the middle cerebral artery. Normal: the bilateral carotid system including M1 is normal.

**Table 3:** CBV, CBF, and MTT measured from normal brain parenchyma of the 30 patients.

Removal	CBV (mL/100 g)	sSVD		cSVD	
		CBF (mL/100 g/min)	MTT (sec)	CBF (mL/100 g/min)	MTT (sec)
None	4.95 ± 0.87	55.3 ± 9.5	6.39 ± 0.70	52.3 ± 8.3	6.04 ± 0.45
CSF	4.81 ± 0.73	56.4 ± 8.6	5.60 ± 0.61*	53.0 ± 6.9	5.42 ± 0.34*
Vessel	3.09 ± 0.24*	36.4 ± 3.7*	6.32 ± 0.67	36.1 ± 2.8*	5.86 ± 0.44
Both	3.11 ± 0.22*	38.7 ± 3.8*	5.49 ± 0.57*	38.0 ± 2.5*	5.21 ± 0.31*

All data are expressed as the means ± standard deviations.

*Significant difference between the original data and data after CSF removal, after vessel removal, and after removal of both (*P < 0.001 from the Mann-Whitney *U* test).
